# Supplementary material for: Applying historical data in a nonlinear mixed-effects model can reduce the number of control rats required for calculation of the relative potency of insulin analogues
Source: PLoS One. 2022 Jun 16;17(6):e0257750. doi: 10.1371/journal.pone.0257750 (PMC9202954; doi:10.1371/journal.pone.0257750)
Supplement: S1 Appendix — (PDF) [file pone.0257750.s001.pdf]

Appendix A: R-code for the different modelling approaches

A.1 Parameter estimation

To obtain reasonable start guesses for the parameters, we have implemented the following function for the initial start guesses. This is used when building the mixed-effects model.

```
Init <- function(mCall, data, LHS){
  #Sort the data and find average GIR per dose.
  xy <- sortedXyData(mCall[["x"]], LHS, data)
  #Ensure 3 dose levels to fit the model, as we have 3 parameters.
  if(nrow(xy) < 3) {stop("Too few distinct x values to fit a logistic")}
  #Upper asymptote
  d <- max(abs(xy[, "y"]))
  #50 percent effective dose
  logED50 <- NLSstClosestX(xy, 0.5 * d)
  #Proportional to the slope
  b <- abs(1 / (NLSstClosestX(xy, 0.73 * d) - logED50))

  value <- c(d, logED50, b)
  names(value) <- mCall[c("d", "logED50", "b")]
  value
}
```

where

- *mCall* is a matched call, which connects the parameters in the model with the parameters in the Init function.

- *LHS* is the expression from the left hand side from the model formula in the nls call.
- The *selfStart* functions makes is possible to fit the model by using the function *Init* as start guesses.

The function *Init* is then used together with *nlsList* to obtain several model fits, one per study:

```
#Model specification
 logist <- selfStart(~ d / (1 + exp(-b * (x - logED50))),
                    Init, c("d", "logED50", "b"))


#Specification of predictor (logdose) and the grouping level (study)
 model_list <- nlsList(GIR ~ logist(logDose, d, logED50, b) | study,
                      data = clamp)
```

A.2 The standard approach

The model is fitted with fixed start guesses using the function nls in R:

```
model  <- nls(GIR ~ d /
              (1 + exp(-b * (logDose - logED50HI + dummyA * logp))),
              data = study,
              start = list(b = 1, d = 51, logED50HI = 2, logp = -1))
```

A.3 The mixed-effects meta approach

Removing vehicle (no dose given) and relevel to get HI as reference:

```
clamp2 <- subset(clamp, clamp$compound != "vehikel")
DF <- within(clamp2, compound <- relevel(compound, ref = "HI"))
```

We fit the model by using the selfstarting function from A.1:

```
model_listA <- nlsList(GIR ~ logist(x = logDose, d, logED50HI, b)
                       | study, data = DF)
```

Model fit with independent random effects on logED50HI and d and fixed target effect on b and d:

```
modelA <- nlme(modelListA, data = DF,
               fixed  = list(d ~ dummyHigh, b ~ dummyHigh,
                            logED50HI ~ as.factor(compound)),
               random = pdDiag(list(logED50HI + d ~ 1)),
               start  = c(fixef(modelA)[1], 0,
                          fixef(modelA)[3], 0,
                          fixef(modelA)[2], rep(0, 69)))
```

A.4 The Bayesian approach

```
library(coda)
library(R2jags)
library(R2WinBUGS)

covMat <- vcov(modelA)[1:5, 1:5]          #Cov matrix from final model
covMat[1,1] <- covMat[1,1] + 1.413532^2  #Random variation on d
covMat[5,5] <- covMat[5,5] + 0.1224241^2 #Random variation on logED50HI
prec_matrix <- solve(covMat)              #Inv cov matrix -> prec matrix
est <- fixef(modelA2)[1:5]                #The mean vector is defined
params <- c("b", "d", "logED50HI", "logp", "mu", "sigma")
```

```
 #Model specification in BUGS language for studies with high target.
 model <- function() {
  for(i in 1:N){
    y[i] ~ dnorm (y.hat[i], tau) #Normal likelihood
    y.hat[i] <- (mu[1] + mu[2]) /
                (1 + exp(-(mu[3] + mu[4])*(x1[i] - mu[5] + x2[i] * logp)))
  }

  tau ~ dgamma(.001, .001) #Vague gamma prior
  logp ~ dunif(-3, 3)      #Uniform. prior for relative log-potency

  mu[1:5] ~ dmnorm(est, prec_matrix) #Multivariat prior
  d <- mu[1] + mu[2]
  b <- mu[3] + mu[4]
  logED50HI <- mu[5]
  sigma <- sqrt(1/tau)
}
write.model(model, "jagsbb.txt") #The model is saved
jags.inits <- function(){ list("mu" = est) } #Initial values for chains

#Fitting the model for a chosen study
jags.fit <- jags("jagsbb.txt",                               #Model
                    data = list('y' = study$GIR,                #Data
                                'N' = nrow(study),
                                'est' = est,                    #Prior
```

```
                                  'prec_matrix' = prec_matrix,     #Prior
                                  'x1' = study$logDose,            #Data
                                  'x2' = study$dummyA),            #Data
                                  n.chains = 3, inits = jags.inits,#Default
                                  n.iter = 7000,
                                  parameters.to.save = params)
  jags.fit.mcmc <- as.mcmc(jags.fit)
  estBay1[1] <- summary(jags.fit.mcmc)$statistics[5,1] #Saving logp est
}
```
