# Supplementary material for: Applying historical data in a nonlinear mixed-effects model can reduce the number of control rats required for calculation of the relative potency of insulin analogues
Source: PLoS One. 2022 Jun 16;17(6):e0257750. doi: 10.1371/journal.pone.0257750 (PMC9202954; doi:10.1371/journal.pone.0257750)
Supplement: Attachment — Submitted filename: Response to Reviewers.pdf [file pone.0257750.s003.pdf]

Response to reviewers

The implementation of the revisions below is shown in red in a revised version uploaded of the original manuscript, the “Revised Manuscript with Track Changes”.

Reviewer #1: The use of external controls from similar studies has been a popular topic mainly among statisticians in clinical trials. These statistical methods show the promise of designing more efficient trials, particularly, in settings of small sample size and slow enrolment. Animal studies are limited by sample size due to cost, logistics, or ethical reasons. I commend the authors of this paper for demonstrating the utility of using historical controls in this interesting and important application. The authors have carefully chosen similar historical studies for information borrowing. A nonlinear mixed-effects model was used to assess the dose-response relationship of insulin analogues as compared to human insulin. The relative log-potency was estimated using a meta-analytic approach and a Bayesian approach. Both approaches yielded comparable results and demonstrated that it would be possible to reduce the number of control animals in a study while borrowing this information from historical studies conducted under similar protocols. Adequate guidance and caution has been provided as to when and how this analysis should be conducted. Although the approach and application are sound, I do have some edits and suggestions to further improve the presentation:

1. It would be helpful to the general readers if the authors followed the journal’s citation guideline:
“References are listed at the end of the manuscript and numbered in the order that they appear in the text. In the text, cite the reference number in square brackets (e.g., “We used the techniques developed by our colleagues [19] to analyze the data”). PLOS uses the numbered citation (citation-sequence) method and first six authors, et al.”

Answer: The manuscript has been updated to meet PLOS ONE’s style requirements, including those for file naming and referencing.

2. In accordance with scientific writing, “meta approach” should be spelled out as “meta-analysis approach” throughout the manuscript.

Answer: meta approach changed to meta-analysis approach throughout the manuscript.

3. Page 1, paragraph 3: Please, clarify the sentence “Sufficient similarity between historical and current control data is also a breaking point for.9”

Answer: ”Sufficient similarity between historical and current control data is also a breaking point for (9) changed to ”A large similarity between historical and current control data is clearly an important factor for incorporating historical data successfully”.

4. Section 2: Please, include a sentence explaining why there are 69 analogues from 59 studies.

Answer: Good point. I have added ”In some of the studies, more than one insulin analogue was included, which results in a larger number of insulin analogues compared to number of studies”

5. Page 5, paragraph 1: The parameters are estimated by maximum likelihood or Markov chain Monte Carlo methods ….
Answer: ”The parameters are estimated by maximum likelihood” changed to ”The parameters are estimated by maximum likelihood or Markov chain Monte Carlo methods, respectively”.

6. Page 9: “markov chain monte carlo” should be “Markov chain Monte Carlo”.

Answer: ”markov chain monte carlo (MCMC)” changed to ”Markov chain Monte Carlo (McMC)”.

7. Results from the Bayesian method should be reported as in Table 1.

Answer: The results from the Bayesian method are reported in Table 3, to compare with the meta-analysis approach results from Table 2. The results from Table 1 are from the mixed-effects model, which we base our Bayesian prior on. Please see section 3.2.3 for details, where one can find: ”Concerning the remaining parameters, we use a multivariate normal prior with the estimates from the mixed-effects meta-analysis from Section 3.2.2 as mean vector, and the variance-covariance matrix is found by using the estimated correlation matrix for these parameters.” I hope this clarifies.

8. In the discussion, the authors have commented that it was possible to achieve higher precision by combining studies “due to a high degree of homogeneity across the historical studies”. This is the ideal case and may not be true in other experiments. The degree of heterogeneity need to be assessed and appropriate methods need to be used to discount the effect of this heterogeneity in the parameter estimates. This is an additional advantage of Bayesian methods. Some established methods are power prior, commensurate prior, and meta-analytic predictive prior as discussed in reference [5]. It would be worthwhile to include this information in the discussions.

Answer: We agree that it is beneficial to mention alternative methods for addressing a possible heterogeneity across studies. In the discussion, we have added the following (in red):

Using the standard method as a benchmark, in Section 4 we investigated the possibility of reducing the number of control rats including historical information and using either a mixed-effects meta analysis or a Bayesian approach, where the latter is based on a construction of a model for the historical data. Due to a high degree of homogeneity across the historical studies, both approaches suggest that including the previous studies is beneficial in regard to reducing the number of control rats when conducting a new study. However, both approaches rely heavily on the mixed-effects model to be used, and therefore the importance of building a suitable model and investigating possible heterogeneity across studies must be underlined and assessed if necessary. In our case, the studies are similar giving a high homogeneity, but this might not be true in other experiments. As discussed in [4], some established Bayesian methods to discount the effect of a possible heterogeneity in the parameter estimates are power prior, commensurate prior, and meta-analytic predictive prior.

The Bayesian approach used in this article essentially adapts what is called the meta-analytic-predictive approach in [4], namely exploiting the series of historical studies at hand in order to construct an informative prior. The “robust” extension of this method, that serves to account for data where the difference between historical and current data exceeds the heterogeneity among the historical trials, or a power prior, might have been beneficial to apply in our case. However, these methods require additional parameters, which would have been challenging to pre-define or estimate. The power prior requires a parameter for weighing the historical studies compared to the current study. For a hierarchical data structure with a time series of historical studies, it is not straightforward how to choose or even estimate this parameter from data. This would require different weights depending on the distance in time between the studies, giving the most recent studies the largest weight. The development of parameter estimates over time in our series of studies has been monitored, see Fig 3, which would also be needed in other experiments, in order to be aware of a possible larger heterogeneity as time passes. If information from some historical studies is not “accurate” in the sense that outlier studies are observed, it should be handled carefully, in order to justify the quality and effects for making valid statistical inference based on this historical information. This could be done by removing or down-weighing some studies (after careful consideration), for example by implementing a variant of a power-prior, using different pre-defined weights.

Reviewer #2: In this paper, the authors investigated the possibility of reducing the number of control rats by using available historical information from the previous studies. Three different procedures within the nonlinear logistic curve framework are considered. Numerical results from simulation studies and real-data application support that the inclusion of historical information is valuable and beneficial. The paper is well-written and I enjoy reading of this paper. My specific comments are as follows.

(1) In this paper, the authors considered both the meta procedure and Bayesian procedure for this study. I wonder if the author consider power prior Bayesian analysis for the current study, in which the power prior is adopted to take the amount of information from historical data into consideration. If so, I strong suggest the authors consider this procedure.

Answer: We have included these considerations in the discussion, see answer to reviewer 1, point 8.

(2) Given that the data is not available and may not publicly available, I wonder how the results will be replicated even though the authors have made the Code available.

Answer: Data is now available as supporting information.

(3) What will happen for the proposed procedures if the historical information is not accurate. In addition, I wonder how the authors deal with the heterogeneity issue of the different studies.
(4) In addition, how do we justify the quality and effects of historical information for making statistical inference quantitatively?

Answer to (3) + (4): This has been addressed in the last part added in the discussion, see point 8: “The development of parameter estimates over time in our series of studies has been monitored, see Fig 3, which would also be needed in other experiments, in order to be aware of a possible larger heterogeneity as time passes. If information from some historical studies is not “accurate” in the sense that outlier studies are observed, it should be handled carefully, in order to justify the quality and effects for making valid statistical inference based on this historical information. This could be done by removing or down-weighing some studies (after careful consideration), for example by implementing a variant of a power-prior, using different pre-defined weights.”

Journal requirements

1. Please ensure that your manuscript meets PLOS ONE's style requirements, including those for file naming. The PLOS ONE style templates can be found at https://journals.plos.org/plosone/s/file?id=wjVg/PLOSOne_formatting_sample_main_body.pdf and https://journals.plos.org/plosone/s/file?id=ba62/PLOSOne_formatting_sample_title_authors_affiliations.pdf

Answer: The manuscript has been updated to meet PLOS ONE's style requirements, including those for file naming.

2. Please update your submission to use the PLOS LaTeX template. The template and more information on our requirements for LaTeX submissions can be found at http://journals.plos.org/plosone/s/latex.

Answer: The submission has been updated with the PLOS LaTex template. Figures, tables and references are in accordance with the requirements in the template.

3. Thank you for stating the following in the Competing Interests section: "I have read the journal's policy and the authors of this manuscript have the following competing interests: Søren Andersen and Christian Lehn Brand are employed by Novo Nordisk and hold shares in the company." Please confirm that this does not alter your adherence to all PLOS ONE policies on sharing data and materials, by including the following statement: "This does not alter our adherence to PLOS ONE policies on sharing data and materials." (as detailed online in our guide for authors http://journals.plos.org/plosone/s/competing-interests). If there are restrictions on sharing of data and/or materials, please state these. Please note that we cannot proceed with consideration of your article until this information has been declared. Please include your updated Competing Interests statement in your cover letter; we will change the online submission form on your behalf.

Answer: The competing interest statement in the cover letter has been updated to "Emilie Prang Nielsen, Søren Andersen and Christian Lehn Brand are employed by Novo Nordisk and hold shares in the company. This does not alter our adherence to PLOS ONE policies on sharing data and materials."

4. In your Data Availability statement, you have not specified where the minimal data set underlying the results described in your manuscript can be found. PLOS defines a study's minimal data set as the underlying data used to reach the conclusions drawn in the manuscript and any additional data required to replicate the reported study findings in their entirety. All PLOS journals require that the minimal data set be made fully available. For more information about our data policy, please see http://journals.plos.org/plosone/s/data-availability. Upon re-submitting your revised manuscript, please upload your study's minimal underlying data set as either Supporting Information files or to a stable, public repository and include the relevant URLs, DOIs, or accession numbers within your revised cover letter. For a list of acceptable repositories, please see http://journals.plos.org/plosone/s/data-availability#loc-recommended-repositories. Any potentially identifying patient information must be fully anonymized. Important: If there are ethical or legal restrictions to sharing your data publicly, please explain these restrictions in detail. Please see our guidelines for more information on what we consider unacceptable restrictions to publicly sharing data: http://journals.plos.org/plosone/s/data-availability#loc-unacceptable-data-access-restrictions. Note that it is not acceptable for the authors to be the sole named individuals responsible for ensuring data access. We will update your Data Availability statement to reflect the information you provide in your cover letter.

Answer: We have received permission to publish the data. I have changed the current data availability statement "Restrictions apply to the availability of this data set, which is available from the authors with the permission of Novo Nordisk" to "The dataset is available as supporting information in S1 Dataset". The cover letter has also been updated with "Data is made available as supporting information".

5. Please include your full ethics statement in the 'Methods' section of your manuscript file. In your statement, please include the full name of the IRB or ethics committee who approved or waived your study, as well as whether or not you obtained informed written or verbal consent. If consent was waived for your study, please include this information in your statement as well.

Answer: The full ethics statement has been inserted in the methods section: "The experimental procedures involving animals were approved by the national Danish Animal Experiments Inspectorate and the Ethical Review Council at Novo Nordisk A/S. The surgical catheter procedure was conducted under general isoflurane anaesthesia followed by three days of analgesia obtained by daily carprofen injections. At the end of the experiments animals were killed by an intravenous overdose of pentobarbital."

6. Please include captions for your Supporting Information files at the end of your manuscript, and update any in-text citations to match accordingly. Please see our Supporting Information guidelines for more information: http://journals.plos.org/plosone/s/supporting-information.

Answer: Captions included for supporting information for S1 Appendix: "R-code for the different modelling approaches" and S1 Dataset: "Dataset analysed in this paper consisting of a time series of 59 glucose clamp studies. The dataset is provided by Novo Nordisk A/S."
